# Reporting and Methods in Clinical Prediction Research: A Systematic Review

**DOI:** 10.1371/journal.pmed.1001221

**Published:** 2012-05-22

**Authors:** Walter Bouwmeester, Nicolaas P. A. Zuithoff, Susan Mallett, Mirjam I. Geerlings, Yvonne Vergouwe, Ewout W. Steyerberg, Douglas G. Altman, Karel G. M. Moons

**Affiliations:** 1 Julius Center for Health Sciences and Primary Care, University Medical Center Utrecht, Utrecht, the Netherlands; 2 Department of Primary Care Health Sciences, University of Oxford, Oxford, United Kingdom; 3 Department of Public Health, Erasmus MC, Rotterdam, The Netherlands; 4 Centre for Statistics in Medicine, University of Oxford, Oxford, United Kingdom; University of Edinburgh, United Kingdom

## Abstract

**Background:**

We investigated the reporting and methods of prediction studies, focusing on aims, designs, participant selection, outcomes, predictors, statistical power, statistical methods, and predictive performance measures.

**Methods and Findings:**

We used a full hand search to identify all prediction studies published in 2008 in six high impact general medical journals. We developed a comprehensive item list to systematically score conduct and reporting of the studies, based on recent recommendations for prediction research. Two reviewers independently scored the studies. We retrieved 71 papers for full text review: 51 were predictor finding studies, 14 were prediction model development studies, three addressed an external validation of a previously developed model, and three reported on a model's impact on participant outcome. Study design was unclear in 15% of studies, and a prospective cohort was used in most studies (60%). Descriptions of the participants and definitions of predictor and outcome were generally good. Despite many recommendations against doing so, continuous predictors were often dichotomized (32% of studies). The number of events per predictor as a measure of statistical power could not be determined in 67% of the studies; of the remainder, 53% had fewer than the commonly recommended value of ten events per predictor. Methods for a priori selection of candidate predictors were described in most studies (68%). A substantial number of studies relied on a *p*-value cut-off of *p*<0.05 to select predictors in the multivariable analyses (29%). Predictive model performance measures, i.e., calibration and discrimination, were reported in 12% and 27% of studies, respectively.

**Conclusions:**

The majority of prediction studies in high impact journals do not follow current methodological recommendations, limiting their reliability and applicability.

*Please see later in the article for the Editors' Summary*

## Abstract 2

Walter Bouwmeester and colleagues investigated the reporting and methods of prediction studies in 2008, in six high-impact general medical journals, and found that the majority of prediction studies do not follow current methodological recommendations.

## Editors' Summary

**Background:**

There are often times in our lives when we would like to be able to predict the future. Is the stock market going to go up, for example, or will it rain tomorrow? Being able predict future health is also important, both to patients and to physicians, and there is an increasing body of published clinical “prediction research.” Diagnostic prediction research investigates the ability of variables or test results to predict the presence or absence of a specific diagnosis. So, for example, one recent study compared the ability of two imaging techniques to diagnose pulmonary embolism (a blood clot in the lungs). Prognostic prediction research investigates the ability of various markers to predict future outcomes such as the risk of a heart attack. Both types of prediction research can investigate the predictive properties of patient characteristics, single variables, tests, or markers, or combinations of variables, tests, or markers (multivariable studies). Both types of prediction research can include also studies that build multivariable prediction models to guide patient management (model development), or that test the performance of models (validation), or that quantify the effect of using a prediction model on patient and physician behaviors and outcomes (impact assessment).

**Why Was This Study Done?:**

With the increase in prediction research, there is an increased interest in the methodology of this type of research because poorly done or poorly reported prediction research is likely to have limited reliability and applicability and will, therefore, be of little use in patient management. In this systematic review, the researchers investigate the reporting and methods of prediction studies by examining the aims, design, participant selection, definition and measurement of outcomes and candidate predictors, statistical power and analyses, and performance measures included in multivariable prediction research articles published in 2008 in several general medical journals. In a systematic review, researchers identify all the studies undertaken on a given topic using a predefined set of criteria and systematically analyze the reported methods and results of these studies.

**What Did the Researchers Do and Find?:**

The researchers identified all the multivariable prediction studies meeting their predefined criteria that were published in 2008 in six high impact general medical journals by browsing through all the issues of the journals (a hand search). They then scored the methods and reporting of each study using a comprehensive item list based on recent recommendations for the conduct of prediction research (for example, the reporting recommendations for tumor marker prognostic studies—the REMARK guidelines). Of 71 retrieved studies, 51 were predictor finding studies, 14 were prediction model development studies, three externally validated an existing model, and three reported on a model's impact on participant outcome. Study design, participant selection, definitions of outcomes and predictors, and predictor selection were generally well reported, but other methodological and reporting aspects of the studies were suboptimal. For example, despite many recommendations, continuous predictors were often dichotomized. That is, rather than using the measured value of a variable in a prediction model (for example, blood pressure in a cardiovascular disease prediction model), measurements were frequently assigned to two broad categories. Similarly, many of the studies failed to adequately estimate the sample size needed to minimize bias in predictor effects, and few of the model development papers quantified and validated the proposed model's predictive performance.

**What Do These Findings Mean?:**

These findings indicate that, in 2008, most of the prediction research published in high impact general medical journals failed to follow current guidelines for the conduct and reporting of clinical prediction studies. Because the studies examined here were published in high impact medical journals, they are likely to be representative of the higher quality studies published in 2008. However, reporting standards may have improved since 2008, and the conduct of prediction research may actually be better than this analysis suggests because the length restrictions that are often applied to journal articles may account for some of reporting omissions. Nevertheless, despite some encouraging findings, the researchers conclude that the poor reporting and poor methods they found in many published prediction studies is a cause for concern and is likely to limit the reliability and applicability of this type of clinical research.

**Additional Information:**

Please access these websites via the online version of this summary at http://dx.doi.org/10.1371/journal.pmed.1001221.

- The EQUATOR Network is an international initiative that seeks to improve the reliability and value of medical research literature by promoting transparent and accurate reporting of research studies; its website includes information on a wide range of reporting guidelines including the REMARK recommendations (in English and Spanish)
- A video of a presentation by Doug Altman, one of the researchers of this study, on improving the reporting standards of the medical evidence base, is available
- The Cochrane Prognosis Methods Group provides additional information on the methodology of prognostic research

## Introduction

In recent years there has been an increasing interest in the methodology of prediction research [1]–[16]. Prediction research includes both diagnostic prediction studies studying the ability of variables or test results to predict the presence or absence of a certain diagnosis, and prognostic studies studying predictors of the future occurrence of outcomes [6],[11],[15]. Both types of prediction research may include single variable (or predictor or test) studies, multivariable studies aimed at finding the independently contributing predictors among multiple candidate predictors, or the development, validation, or impact assessment of multivariable prediction models. Many have stressed the importance of pre-defining the key aspects of a study, including aims, study design, study population, clinically relevant outcomes, candidate predictors, sample size considerations, and statistical analysis. Use of poor methods may lead to biased results [2],[7],[10],[12],[13],[15],[17]–[32].

We performed a comprehensive literature review of articles published in high impact general medical journals to assess whether prediction research in the recent literature was conducted according to methodological recommendations. We considered all types of clinical prediction studies and all methodological issues that are considered to be important in prediction research, rather than on specific types of outcomes [33], specific methodological issues [34], or specific disease areas [20],[21],[35]–[37]. We focus on the reporting of aim, design, study sample, definition and measurement of outcomes and candidate predictors, statistical power and analyses, model validation, and results, including predictive performance measures.

## Methods

### Literature Search

**Figure 1:**
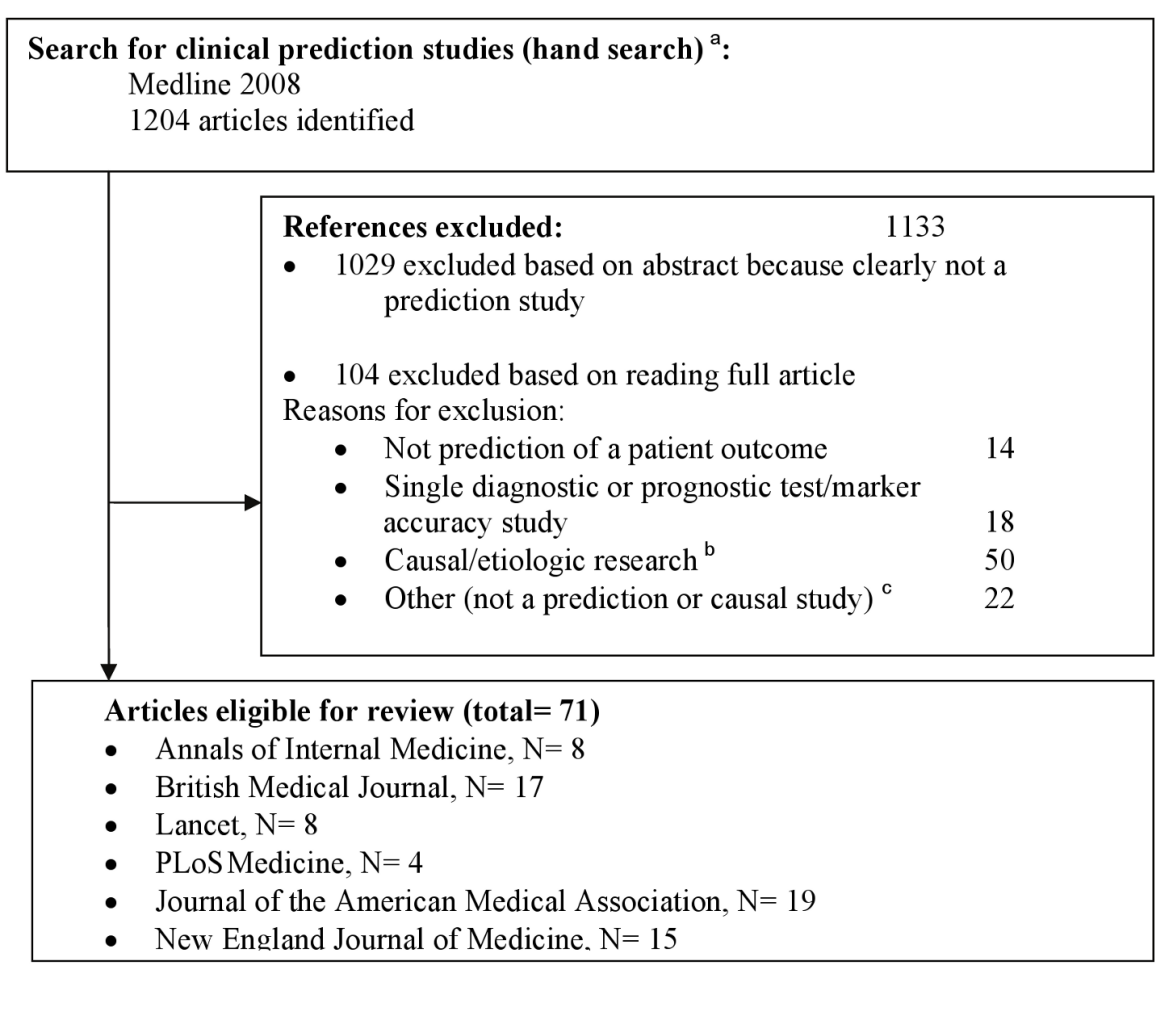
Flowchart of included studies. ^a^The hand search included only studies with an abstract, published in 2008 in *The New England Journal of Medicine*, *The Lancet*, *JAMA: the Journal of the American Medical Association*, *Annals of Internal Medicine*, *BMJ*, and *PLoS Medicine*. The following publication types were excluded beforehand: editorials, bibliographies, biographies, comments, dictionaries, directories, festschrifts, interviews, letters, news, and periodical indexes. ^b^Studies, generally conducted in a yet healthy population, aimed at quantifying a causal relationship between a particular determinant or risk factor and an outcome, adjusting for other risk factors (i.e., confounders). ^c^For example, see [72].

We fully hand searched the six highest impact (based on Web of Knowledge impact factors) general medicine journals for the year 2008 (Figure 1). We excluded all studies that were not original research (e.g., editorials, letters) or had no abstract. One reviewer (W. B.) examined titles and abstracts of citations to identify prediction studies. The full text of all thus selected studies was obtained, and two authors (W. B. and N. P. A. Z.) independently assessed eligibility; in case of doubt a third independent reader was involved (K. G. M. M. or Y. V.).

### Inclusion Criteria

We focused on multivariable prediction studies that were defined as descriptive studies where the aim was to predict an outcome by two or more independent variables, i.e., a causal relationship between independent variable(s) and outcome was not necessarily assumed [6],[11]. We included both diagnostic and prognostic multivariable prediction studies. We excluded studies that investigated a single predictor, test, or marker (such as single diagnostic test accuracy or single prognostic marker studies), studies that investigated only causality between one or more variables and an outcome, and studies that could not contribute to patient care, e.g., predictor finding studies to predict citation counts.

### Development of Item List

We developed a comprehensive item list based on the existing methodological recommendations for conducting and reporting prediction research, and on extensive discussions among the co-authors. To this aim we studied existing reporting statements and checklists (e.g., CONSORT, REMARK, STARD, and STROBE) and quality assessment tools from other domains (e.g., QUADAS) for those aspects that also pertain to multivariable prediction studies, e.g., study aims, design, and participant selection [4],[38]–[41]. Further, to identify additional aspects relevant for good conducting and reporting of multivariable prediction research, we consulted published recommendations for prediction research and the references of these studies [1]–[10],[12]–[16],[20],[21],[27],[28],[42]–[44].

### Data Extraction

Data were extracted to investigate both the reporting of and use of methods known to influence the quality of multivariable prediction studies. The main items that we extracted are summarised in Box 1. For investigation of statistical power in prediction studies, we considered the individual multivariable models within studies, because power differed among models within a single study.

**Box 1. Overview of Items Addressed in This Review**

**Study design**

Type of prediction study (e.g., model development); participant sampling or selection method (e.g., cohort, case-control approach)

**Participants**

Participant recruitment; follow-up; inclusion and exclusion criteria; setting (e.g., primary or secondary care or general population)

**Candidate predictors**

Clear definition to ensure reproducibility; coding of predictor values; assessment blinded for outcome

**Outcome**

Clear definition to ensure reproducibility; type of outcome; assessment blinded for predictors

**Statistical power**

Effective sample size (e.g., number of outcome events compared to number of candidate predictors)

**Selection of predictors**

Selection of predictors prior to statistical analysis and within statistical analysis; use of variable selection strategies (e.g., backward selection); criterion for predictor inclusion (e.g., *p*<0.05)

**Handling of missing values**

Reporting of missing values per predictor, or number or percentage of participants with missing values; reporting of procedures for dealing with missing values

**Presentation of results**

Reporting of univariable and multivariable predictor–outcome effects; reporting of full or final model

**Model performance measures and validation**

Type of predictive performance measures reported (e.g., *C*-statistic and calibration); type of validation (e.g., internal or external)

Items were scored as present, absent, not applicable, or unclear. If an item concerned a numeric value (e.g., the number of participants) we extracted this value. If a description was unclear, we classified it as not described or separately reported it in our tables. If studies referred to other papers for detailed descriptions, the corresponding items were checked in those references.

Two authors (W. B., N. P. A. Z.) independently extracted the data. In case of doubt, items were discussed with a third and fourth reviewer (K. G. M. M., Y. V.). The inter-reviewer agreement on the data extraction was assessed by calculating the percentage of overall agreement between the two reviewers.

### Analysis

We distinguished five types of multivariable prediction research.

#### Predictor finding studies

These studies aim to explore which predictors out of a number of candidate predictors independently contribute to the prediction of, i.e., are associated with, a diagnostic or prognostic outcome [3],[6],[28].

#### Model development studies without external validation

These studies aim to develop a multivariable prediction model, e.g., for use in practice to guide patient management. Such studies aim to identify the important predictors, assign the (mutually adjusted) weights per predictor in some kind of multivariable analysis, and develop a final multivariable prediction model [6],[7]. These studies might include internal validation studies, such as random split-sample methods, cross-validation, or bootstrapping [43].

#### Model development studies with external validation

These studies have the same aim as the previous type but also test the performance of the developed model in a so-called external dataset from, e.g., another time period (temporal validation) or another hospital, country, or setting (geographical validation). Explicit withholding of the data from some study centres for validation was also considered as (geographical) external validation.

#### External validation studies without or with model updating

These studies aimed to assess the performance of a previously reported prediction model using new participant data that were not used in the development process, and in some cases adjusted or updated the model based on the validation data when there was poor validation [8]–[10],[45]–[47].

#### Model impact studies

These studies aim to quantify the effect or impact of using a prognostic or diagnostic prediction model on patient or physician behaviour and management, patient health outcomes, or cost-effectiveness of care, relative to not using the model [9],[45],[48].

We grouped results by type of multivariable prediction research, medical specialty (oncology, cardiovascular diseases, other), and whether the prediction analysis was a primary or secondary study aim.

### Ethics Statement

An ethics statement was not required for this work.

## Results

We identified 1,204 articles by hand searching, of which 71 met the inclusion criteria (Figure 1 and Text S2). Most studies were excluded based on title or abstract. It was difficult to distinguish, from study abstracts alone, between descriptive predictor finding studies and articles studying causality of a factor. We thus read 104 full text papers, excluding 50 studies deemed causal (Figure 1). The PRISMA checklist is provided as Text S1.

### Data Extraction and Reviewer Agreement

The two reviewers agreed on a median of 92% (interquartile range 75%–100%) of the items extracted. Most discrepancies related to specific participant sampling strategies or participant sources and were resolved after discussion with a third reviewer.

The main challenge was to distinguish between predictor finding and model development studies. Authors in general did not explicitly state their aim, so we used full text interpretations to classify studies as predictor finding or model development studies.

### Study Aims

Most multivariable prediction studies were published in the field of cardiovascular diseases (*n* = 24) (Table 1). The aim was usually to identify independently contributing predictors of an outcome (*n* = 51/71). Of the prediction modelling studies (*n* = 20), the vast majority were model development studies, without (*n* = 11) or with (*n* = 3) external validation. Pure external validation (*n* = 3) and impact studies (*n* = 3) were rare. There were few multivariable diagnostic studies (*n* = 5). In the 71 publications, 135 models or sets of predictors were studied. For example, in predictor finding studies a search for independently contributing predictors might be applied across different participant subgroups (e.g., males versus females) for multiple outcomes, and in prediction modelling studies, more than one model might be developed or validated (e.g., for different outcomes or presenting a basic and extended model).

**Table 1:** Aim of the included multivariable prediction studies, subdivided by clinical domains.

Study Aim	Cardiovascular (*n* = 24)	Oncology (*n* = 13)	Other^a^ (*n* = 34)	Total Papers (*n* = 71)	Number of Models (*n* = 135)	Number of Diagnostic Studies
**Predictor finding studies**						
Prediction was primary aim	46 (11)	62 (8)	44 (15)	48 (34)	49 (66)	1
Prediction was secondary aim	17 (4)	31 (4)	26 (9)	24 (17)	21 (28)	0
**Prediction model development without external validation**	21 (5)	8 (1)	15 (5)	15 (11)^b^	14 (19)	1
**Prediction model development with external validation**	4 (1)	0 (0)	6 (2)	4 (3)	8 (11)	0
**External validation, without updating a prediction model** b	8 (2)	0 (0)	3 (1)	4 (3)	5 (7)	1
**Impact assessment of a prediction model**	4 (1)	0 (0)	6 (2)	4 (3)	3 (4)	2

Numbers are column percentages, with absolute numbers in parentheses.

a Including studies from infectious diseases (*n* = 7), diabetes (*n* = 5), neonatology and child health (*n* = 6), mental disorders (e.g., dementia) (*n* = 4), and musculoskeletal disorders (e.g., lower back pain) (*n* = 4).

b There were no external validation studies of a previously published model that also updated the model after poor validation.

### Design of Participant Sampling

A cohort, nested case-control, or case-cohort design is commonly recommended for prognostic and diagnostic model development and validation [6]. A prospective cohort is preferable, because it enables optimal measurement of predictors and outcome. A retrospective cohort may allow for a longer follow-up period but usually at the expense of poorer data [6]. Randomized trial data have advantages similar to those of prospective cohort data, unless restrictive eligibility criteria make the study sample unrepresentative. Further, treatments proven to be effective in the trial should be included or adjusted for in the modelling. Cohort, nested case-control and case-cohort datasets each allow for calculation of absolute outcome risk [6],[18],[49]. A non-nested case-control design may, however, be sufficient for predictor finding studies, since these studies generally do not aim to calculate absolute risks.

We found that case-control designs were indeed used only by predictor finding studies (Table 2). Prospective cohort data, either observational or randomized trial data, were most frequently used (*n* = 44). Quantifying the impact of a prediction model on participant outcome requires a comparative study design [9],[48]. A randomized trial was used by two of the three impact studies; the third used an observational before-after (prospective) cohort design, comparing participant outcomes before and after the introduction of a prediction model.

**Table 2:** Study design in relation to study aim.

Study Design	Total (*n* = 71)	Predictor Finding Studies (*n* = 51)	Development without External Validation (*n* = 11)	Development with External Validation (*n* = 3)	External Validation (without Updating) (*n* = 3)	Impact Analysis (*n* = 3)	Specifications (*n*)
**Prospective cohort** a	62 (44)	53 (27)	82 (9)	100 (3)	67 (2)	100 (3)	Cross-sectional (1)
							Randomized trial (13)^b^
**Retrospective cohort** a	14 (10)	16 (8)	9 (1)	0 (0)	33 (1)	0 (0)	Cross-sectional (2)
**Case-control** c	8(6)	12 (6)	0 (0)	0 (0)	0 (0)	0 (0)	Nested (4)
							Non-nested (2)
**Not described or unclear**	15 (11)	20 (10)	9 (1)	0 (0)	0 (0)	0 (0)	

Numbers are column percentages, with absolute numbers in parentheses, except for the column “Specifications”, which includes only absolute numbers.

a Some cohort studies had a cross-sectional cohort design, which was possible because the predictor values did not change (gender, genes, etc.) or because the study involved a diagnostic prediction model study.

b Of the 13 studies that used randomized trial data, 11 were predictor finding or model development studies. Of these 11 studies, five adjusted for the treatment effect, three did not adjust because there was no treatment effect, one did not adjust despite an effective treatment, and in two studies reporting and adjustment for treatment effects was entirely missing.

c One study used two designs: a cross-sectional case-cohort and a cross-sectional nested case-control (here both scored as nested case-control).

### Participant Recruitment, Follow-Up, and Setting

Participant recruitment was in general well described. Inclusion criteria were reported in 64/71 studies. Description of the cohort characteristics was clear in 68/69 of the relevant prediction studies (not applicable for two non-nested case-control studies). Study recruitment dates were reported in 88% of the studies. Length of follow-up was not reported in nine studies, leaving readers unable to know the time period for the predicted risks of the models. Whether (all) consecutive participants were included or how many participants refused to participate was rarely reported and so could not be evaluated. The majority of studies involved participants from a hospital setting (38%) or the general (healthy) population (27%). Clinical setting was not reported in 4% of the studies.

### Outcome

In outcome reporting, we expected differences between studies with prediction as a primary versus a secondary aim, but this was not observed. Outcomes were well defined in 62/68 studies (Table 3). However, only 12 studies reported that they blinded the outcome measurement for predictor values. Knowledge of the predictors might influence outcome assessment, resulting in a biased estimation of the predictor effects for the outcome [6],[17],[39]. 11/68 studies had all cause mortality as the outcome, where such bias would not be a factor.

**Table 3:** Reporting of outcomes.

Reporting and Analysis of Outcomes	Percentage (*n*)
**Clear definition**	91 (62)
**Assessment blinded for predictors** a	22 (12)
**Type of outcome described** b	93 (63)
**Continuous**	9 (6)
Linear regression	83 (5)
Logistic regression^c^	17 (1)
**Binary**	34 (23)
Logistic regression	91 (21)
Non-regression^d^	9 (2)
**Categorical**	12 (8)
Polytomous regression	38 (3)
Logistic regression	50 (4)
CART	13 (1)
**Time to event**	48 (30)
Survival analysis	97 (29)
Logistic regression	3 (1)

Impact studies were excluded from this table because these studies had outcomes of a different type (e.g., costs). Hence, the total number of studies is 68.

a Not applicable in 11/68 studies, because all cause death was the outcome.

b Types of outcomes and how they were analysed (unclear for five studies). The sum 6+23+8+30 is higher than 63 because some outcomes were analysed in more than one way (e.g., a time-to-event outcome that was analysed as time to event and as a binary outcome neglecting time). If a study analysed two binary outcomes, it was here counted as one binary outcome.

c After dichotomization of a continuous outcome.

d One study used the Cochran–Mantel–Haenszel procedure, another calculated odds ratios.

CART, classification and regression tree.

Most studied outcomes were binary or time-to-event outcomes. Some outcomes are binary per se, but in some studies, continuous, categorical, and time-to-event data were analyzed as binary outcomes, a practice that is not recommended because less accurate predictions are likely to result, as with dichotomizing predictor variables [50].

Prediction of more than one outcome was very common in predictor finding studies, apparently because of their exploratory aim (Table S1). However, selective reporting of outcomes (and predictors) is often a risk [51]. Unfortunately, study registration is not mandated for prediction research, so it is generally impossible to assess whether some outcomes were analysed but not reported.

A number of studies predicted a combined endpoint (14/71). The use of a combined endpoint will give problems if the predictor effect is in opposite directions for different outcomes included in the composite endpoint [52],[53].

### Candidate Predictors

Description of the candidate predictor variables was in general clear (59/68) (Table 4). In 51 of the 68 studies, predictor measurement was blinded for the outcome: in 44, simply because of the prospective design; in seven non-prospective studies, predictor measurement was explicitly blinded for the outcome. One study also assessed the predictors independently, i.e., the predictors that were studied for whether they added value to an existing model were assessed without knowledge of the predictors in that model. Predictor interaction (non-additivity) was tested in 25 of the 51 predictor finding studies and in 11 of the 14 model development studies (total *n* = 36). Dichotomization of continuous predictors is still common practice (21/64), despite being discouraged for decades [3],[50].

**Table 4:** Reporting of candidate predictors.

Reporting and Handling of Candidate Predictors	Percentage (*n*)
**Clear definition**	87 (59)
**Assessment blinded for outcome(s)**	75 (51)
**Predictor part of outcome**	1 (1)
**Interaction of predictors tested** a	55 (36)
**Handling of continuous predictors described** b	67 (43)
Kept linear (continuous)	67 (43)
(Fractional) polynomial transformation or any spline transformation	19 (12)
Categorised	47 (30)
Dichotomized	33 (21)
Other	3 (2)

Impact studies (*n* = 3) were excluded from this table as their aim is not to develop or validate a prediction model, but rather to quantify the effect or impact of using a prediction model on physicians' behaviour, patient outcome, or cost-effectiveness of care relative to not using the model or usual care. Hence, for this table total *n* = 68.

a Not applicable for the three external validation studies. Hence, *n* = 65.

b Not applicable in four studies, because one studied no continuous predictors, and the others were the three external validation studies. Hence, *n* = 64. Of these, handling was unclear in 19 studies, not described in two studies. The sum 43+12+30+21+2 is more than 43 because some studies handled continuous predictors in two ways (e.g., dichotomizing blood pressure and categorising body mass index into four categories).

### Statistical Power

For assessment of statistical power in studies estimating predictor effects for binary or categorical event outcomes, the number of participants in the smallest group determines the effective sample size. A frequently mentioned rule of thumb is “10 events needed per candidate predictor” [12],[25],[26],[30],[54]. For time-to-event outcomes, the effective sample size is also highly related to the number of participants who experience the event [12]. For continuous outcomes, the effective sample size is determined by the number of participants included in the linear regression analysis.

The number of candidate predictors should include all variables initially considered in the study as potential predictors, and not only those considered or included in the multivariable analysis. The candidate predictors also include the number of predictor interactions tested and the number of dummy variables used to include a categorical predictor in a model.

For predictor finding and model development studies, we calculated the statistical power of the fitted models based on (1) the number of predictors eventually included in the final model and (2) the number of candidate predictors (Table 5). Based on the former, as expected, the statistical power was indeed >10 events per variable in 84 (11+60+1+13) of the 124 (96+28) models fitted in these studies. However, there was insufficient reporting of the number of candidate predictors in the vast majority of these studies, such that a proper estimation of the statistical power could not be made. In the studies that clearly described the number of candidate predictors, the effective sample size was <10 events per variable in 50% (*n* = 21) of the presented models.

**Table 5:** Effective sample size of the included studies (reflecting statistical power).

Effective Sample Size	Prediction as Primary Aim (*n* = 96 Models)^a^	Prediction as Secondary Aim (*n* = 28 Models)^a^
**Considering only the predictors in the final model**		
<5	8 (8)	0 (0)
5–10	6 (6)	25 (7)
10–15	11 (11)	4 (1)
>15	63 (60)	46 (13)
Number of participants or events not described	11 (11)	25 (7)
**Considering all candidate predictors** b		
<5	7 (7)	14 (4)
5–10	7 (7)	11 (3)
10–15	0 (0)	0 (0)
>15	19 (18)	11 (3)
Number of candidate predictors not described	67 (64)	64 (18)

Numbers are column percentages, with absolute numbers in parentheses. For continuous outcomes, the effective sample size is the number of participants divided by the number of predictors; for dichotomous outcomes, the effective sample size is the number of participants in the smallest category divided by the number of predictors; for time-to-event outcomes, the effective sample size is the number of events divided by the number of predictors.

a Excluding impact and external validation studies, because they require very different statistical power calculations.

b The number of candidate predictors was the total number of degrees of freedom (i.e., the sum of all candidate predictors, interactions, and dummy variables).

To externally validate a prediction model, a minimum effective sample size of 100 participants with and 100 without the event has been recommended [55]. Given this, effective sample size was sufficient in the majority of the external validation studies (9/13 models).

Across all 71 included prediction studies, only 12 gave an explicit sample size calculation.

### Selection of Candidate and Final Predictors

Adequate reporting of predictor selection methods used is important, because the number of candidate predictors and how they were selected at various stages of the study can both influence the specific predictors included in the final multivariable model, and thus affect the interpretation of the results 12,13,43,56. This issue is not specific to prediction studies but also arises in causal research, although here variables to be included in the multivariable modelling are usually referred to as confounders. Ideally, candidate predictors are selected based on theoretical or clinical understanding. There is no clear cut method that is widely recommended to select independent variables from candidate variables. However, many methodological reports have shown that selection based on (significant) univariable predictor–outcome associations is not recommended, as this method increases the chance of biased results in terms of spurious predictors and overfitted and unstable models [12],[13],[43],[56]. In multivariable analyses, predictors are most often selected based on backward or forward selection, typically using a significance level of 0.05. However, the use of multivariable selection methods can also lead to overfitting and unstable models, especially when there are relatively few outcome events and many predictors analysed [10],[12],[13].

We found that selection of candidate predictors was described for 36 (75%) of the studies where prediction was the primary aim and for eight (47%) of the studies where prediction was a secondary aim (Table 6). In studies with prediction as the primary aim, the majority (71%) selected their candidate predictors based on existing literature, whereas this was less often the case (29%) in studies with prediction as a secondary aim.

**Table 6:** Method of predictor selection, stratified by whether prediction was the primary or secondary study aim.

Selection Method	Prediction as Primary Aim (*n* = 48)	Prediction as Secondary Aim (*n* = 17)	Total (*n* = 65)
**Selection of predictors for inclusion in the multivariable analysis**			
**Not based on statistical analysis** a **^,^** b			
Method described	75 (36)	47 (8)	68 (44)
Literature based	71 (34)	29 (5)	60 (39)
A priori hypothesis/clinical reasoning	29 (14)	29 (5)	29 (19)
**Based on statistical analysis**			
Screening by univariable analysis	13 (6)	24 (4)	15 (10)
**Method of predictor selection used within multivariable analysis** a			
Backward selection	17 (8)	18 (3)	17 (11)
Forward selection	6 (3)	0 (0)	5 (3)
Added value of a specific predictor to existing predictors or model^c^	25 (12)	0 (0)	18 (12)
All predictors included regardless of statistical significance	40 (19)	47 (8)	42 (27)
Similar predictors combined^d^	17 (8)	6 (1)	11 (7)
Method not described	27 (13)	35 (6)	29 (19)
**Criterion for selection of predictors in multivariable analyses** e			
*p*-Value cut-off at <0.05 or lower	21; 29 (10)	12; 18 (2)	18; 26 (12)
*p*-Value cut-off higher than 0.05	4; 6 (2)	12; 18 (2)	6; 9 (4)
Akaike's Information Criterion	4; 6 (2)	0; 0 (0)	3; 4 (2)
Bayesian Information Criterion	2; 6 (1)	6; 9 (1)	3; 4 (2)
Explained variance (*R* ^2^)	4; 6 (2)	0; 0 (0)	3; 4 (2)
Change in *C*-statistic	10; 14 (5)	0; 0 (0)	9; 13 (6)

Numbers are column percentages, with absolute numbers in parentheses. Impact and external validation studies (*n* = 6) were excluded from this table as these issues are not applicable for these type of studies. Hence, *n* = 65.

a More than one method may be used within a study; percentages do not add up to 100%.

b Percentage (number) of studies that reported the applied method for selecting which predictors were included in the multivariable analyses, if it was not based on statistical analysis (i.e., univariable predictor–outcome associations).

c Predictor inclusion in multivariable model was pre-specified, as the specific aim was to quantify the added value of a new predictor to existing predictors.

d For example, systolic and diasystolic blood pressure combined to mean blood pressure.

e For the items below, two percentages are given. The first percentage includes all studies (i.e., 48 predictor finding studies, 17 model development studies, or 65 total); the second is the percentage of all studies that applied some type of predictor selection in the multivariable analysis (35 predictor finding studies, 11 model development studies, and 46 total; the excluded studies did not apply any predictor selection in the multivariable analysis but simply pre-specified the final model).

Pre-selection of candidate predictors for inclusion in the multivariable analyses based on univariable predictor–outcome associations was used in 13% of the primary-aim and in 24% of the secondary-aim prediction studies.

The method of selection of predictors within multivariable models was not described in 19% of the studies. Studies reported using backward selection in 17% of the primary-aim and 18% of the secondary-aim studies, whereas forward selection was reported in 6% and 0%, respectively. 18% of all studies investigated the added value of specific predictors, and 42% included predictors regardless of statistical significance.

The most commonly reported criterion for predictor selection in multivariable models was a *p*-value of <0.05, used in 18% of all studies (and in 26% of the studies that indeed applied predictor selection in multivariable analyses). Other criteria, such as Akaike's Information Criterion or *R* ^2^, were used much less frequently.

### Missing Values

Missing values in clinical study data rarely occur completely at random. Commonly missing values are related to observed participant or disease characteristics. Exclusion of participants with missing values will therefore not only lead to loss of statistical power, but often also to biased results 10,12,13,23,24,57,58. Imputation, notably multiple imputation, of missing values is often advocated to preserve power and obtain less biased results, on the assumption that the reason for the missing data is not entirely due to non-observed information (i.e., data are not “missing not at random”). When there are few missing observations, for example <5% of the individual values in the data, sometimes simple methods are advocated such as single imputation or imputation of the mean [13],.

Occurrence and handling of missing values was not described or was unclear in 38% of all studies (Table 7). If reported, it was mostly reported by “missing values per predictor” (58%). Loss to follow-up was reported in 46% of the studies where this was applicable.

**Table 7:** Handling of missing values, stratified by whether prediction was the primary or secondary study aim.

Reporting and Handling of Missing Values	Prediction as Primary Aim (*n* = 48)	Prediction as Secondary Aim (*n* = 17)	External Validation Studies (*n* = 3)	Impact Studies (*n* = 3)	Total (*n* = 71)
**Reporting of missing data** a					
Not reported or unclear	35 (18)	53 (9)	0 (0)	0 (0)	38 (27)
Number of participants with missing values	23 (11)	12 (2)	67 (2)	0 (0)	21 (15)
Number of missing values per predictor	60 (29)	47 (8)	33 (1)	100 (3)	58 (41)
Number lost to follow-up^b^	40 (16)	50 (7)	50 (1)	100 (3)	46 (27)
**Methods used for handling of missing data** c					
Complete case analysis^d^	71 (33)	53 (9)	67 (2)	33 (1)	65 (45)
Predictor with missing values omitted	2 (1)	12 (2)	0 (0)	0 (0)	4 (3)
Missing indicator method	14 (7)	12 (2)	0 (0)	0 (0)	13 (9)
Single imputation	2 (1)	6 (1)	0 (0)	0 (0)	3 (2)
Multiple imputation	10 (5)	0 (0)	0 (0)	0 (0)	7 (5)
Sensitivity analysis^e^	6 (3)	24 (4)	0 (0)	0 (0)	10 (7)
Not reported or unclear	50 (23)	65 (11)	33 (1)	67 (2)	54 (37)

Numbers are column percentages, with absolute numbers in parentheses.

a Some studies reported more than one item. Hence, percentages do not add up to 100%.

b Cross-sectional studies were excluded for this item (item not applicable).

c More than one method could be applied. Hence, the percentages do not add up to 100%. Items were not applicable for two primary-aim studies that had no missing values. Hence, total *n* = 69.

d Only participants with completely observed data were analysed.

e For example: in a diagnostic study [73], the investigators assumed that among participants who did not undergo follow-up colonoscopy, the detection rates for any adenoma and for an advanced adenoma ranged from half to twice the rates among participants who did undergo follow-up colonoscopy.

Analysis of participants with complete data (i.e., complete case analysis) was performed in the vast majority of studies. It is likely that the studies that did not or unclearly reported the method of handling missing values applied a complete case analysis as well. By comparison, multiple imputation, the most rigorous strategy for dealing with missing values, was used in only 8% of all studies. With the missing indicator method, a dummy or indicator (0/1) variable is created for every predictor with missing values, with 1 indicating a missing value for the original predictor and 0 indicating an observed value. This predictor is then included as a separate predictor in the multivariable analysis. Even though this method is known to lead to biased results in almost all observational studies [13],[23],[60]–[62], it was still used in 13% of the studies investigated here.

### Presentation of Results

Most guidelines, such as the STROBE guidelines for the reporting of observational studies or the REMARK guidelines for tumour marker prognostic studies, specifically advise investigators to report both unadjusted results (i.e., from univariable analysis, yielding the association of each candidate predictor with the outcome) and adjusted results (i.e., from a multivariable analysis) [4],[38],[63]. Presenting results from both analyses allows readers insight in the predictor selection strategies and allows them to determine the influence of the adjustment for other predictors. For prediction studies that apply predictor selection methods in the multivariable analyses, the presentation of a “full” model, a model that includes all predictors considered, may therefore be valuable.

Few studies reported adjusted (20%) or unadjusted (18%) results of the full model with all candidate predictors considered (Table 8). The majority, 65% of the predictor finding and 79% of the model development studies, reported the predictor coefficients or effect estimates of the model after predictor selection (the final model).

**Table 8:** Presentation of the results, stratified by type of prediction study.

Type of Result Presented	Predictor Finding Studies (*n* = 51)	Development Studies (*n* = 14)	Total (*n* = 65)^a^
Unadjusted (univariable) candidate predictor-outcome association	18 (9)	21 (3)	18 (12)
Unadjusted association only of the predictors eventually included in the final model(i.e., after predictor selection)	37 (19)	29 (4)	35 (23)
Adjusted associations of each predictor in full multivariable model	18 (9)	29 (4)	20 (13)
Adjusted associations of each predictor in final multivariable model	65 (33)	79 (11)	68 (44)
Simplified risk score/nomogram/score chart	4 (2)	36 (5)	11 (7)

Numbers are column percentages, with absolute numbers in parentheses. Impact and external validation studies (*n* = 6) were excluded from this table as these items were not applicable. Hence, total *n* = 65.

a The percentages do not add up to 100%, because studies reported univariable and multivariable models. Further, all studies reporting the full model also reported the final model.

### Model Performance and Internal and External Validity

The assessment of the predictive performance of a prediction model is important for understanding how predictions from the model correspond to the observed outcomes. Predictive performance of a model can be assessed on the same data that was used to generate the results (referred to as the apparent performance in the development dataset), or in random (cross-validated) subsamples of the development dataset, or using resampling techniques (like bootstrapping), all referred to as internal validation of the performance of the prediction model [2],[8],[10],[48],[64],[65]. Quantifying or validating a model's predictive performance in new subject data (i.e., subjects other than those used for the model development or internal validation) is the most rigorous form of model validity assessment and is referred to as external validation [8],[10],[32],[64],[66].

In prediction research, two main types of prediction performance measures are usually distinguished: calibration, which is the agreement between predicted outcome and observed outcome, and discrimination, which is the ability to separate participants with and without the outcome of interest [13],[67]. In addition, overall measures for discrimination and calibration (e.g., the *R* ^2^ and Brier scores) may also be reported.

Calibration was reported in only a few studies (Table 9). If done, the Hosmer-Lemeshow statistic was the most often reported calibration measure. Discrimination was assessed with the *C*-statistic or area under the receiver operating characteristic (ROC) curve in 12% of the predictor finding and, as expected, 80% of the model development and external validation studies. *R* ^2^ and Brier score were reported in very few studies. Internal validation was performed in 33% (*n* = 5) of the 14 model development studies, and external validation in only four studies.

**Table 9:** Model performance measures, stratified by type of prediction study.

Performance measure	Predictor Finding Studies (*n* = 51)	Development (*n* = 14) and External Validation (*n* = 1) Studies Combined^a^	Total (*n* = 66)^a^
**Calibration measures**			
Calibration plot	0 (0)	27 (4)	6 (4)
Calibration intercept and slope	0 (0)	0 (0)	0 (0)
Hosmer-Lemeshow statistic	4 (2)	27 (4)	9 (6)
**Discrimination measures**			
*C*-statistic/AUC-ROC	12 (6)	80 (12)	27 (18)
**Classification**			
NRI	2 (1)	40 (6)	11 (7)
Sensitivity/specificity	2 (1)	7 (1)	3 (2)
Other	2 (1)	33 (5)	9 (6)
**Overall performance measures**			
Brier score	0 (0)	7 (1)	2 (1)
*R* ^2^	8 (4)	13 (2)	9 (6)
**Validity assessment**			
Apparent^b^	18 (9)	60 (9)	27 (18)
Internal with jack-knife	0 (0)	7 (1)	2 (1)
Internal with (random) split sample	0 (0)	13 (2)	3 (2)
Internal with bootstrapping techniques	4 (2)	13 (2)	6 (4)
External	0 (0)	27 (4)	6 (4)

Numbers are column percentages, with absolute numbers in parentheses. The percentages sometimes do not add up to 100% because development studies commonly reported more than one performance measure or validity assessment.

a Impact studies (*n* = 3) were excluded since all items were not applicable. Additionally, two external validation studies were excluded because they evaluated risk stratification tools that did not provide predicted probabilities (the Manchester triage system [74] and predictive life support tools [75]). Hence, almost all items were not applicable. Hence, for this table total *n* = 66 studies.

b The predictive performance (e.g., *C*-statistic, calibration, or net reclassification index) of the prediction model as estimated from the same data from which the model was developed.

AUC-ROC, area under the receiver operation characteristic curve; NRI, net reclassification index.

## Discussion

We have described the state of current prediction research, and highlighted aspects that clearly need improvement. We assessed the reporting and methods in all clinical prediction studies published in six high-impact general medical journals in 2008. Our investigation found that among the 71 prediction studies identified, the vast majority were predictor finding studies (*n* = 51), followed by model development studies (*n* = 14). External validation and model impact studies were rare (*n* = 6). Study design, participant selection, definitions of outcomes and predictors, and predictor selection were generally well reported. However, improvements are clearly needed, both in conduct and in reporting of the following: how predictors and outcomes are assessed (with a focus on mutual blinding); the handling of continuous predictors; whether predictor interactions are studied; statistical power and effective sample size considerations; occurrence and handling of missing data; the presentation of the results in both the univariable and multivariable analysis; and the methods used to quantify and notably validate the predictive performance of prediction models.

We found that 14 studies developed new prediction models (of which three included an external validation). Three studies externally validated models, and three investigated the impact of an existing prediction model. Various reports have indicated that in prediction modelling research there is an unfortunate practice of developing new models instead of externally validating or updating existing models [8]–[10],[15],[29],[36],[48]. However, we found a similar number of model development studies (*n* = 14), and studies that aimed to evaluate an existing model (*n* = 6+3).

We do acknowledge that various basic items are well described and reported in prediction studies, for example aim, participant selection, inclusion and exclusion criteria, and design. These items have been identified as important in several well-known guidelines for reporting of clinical research [4],[38]–[41]. Journals systematically, and apparently effectively, refer to these guidelines in their “instructions for authors”. However, sample size considerations, applied statistical methods, and procedures for dealing with missing data were poorly reported despite being highlighted in several reporting guidelines. Poor reporting of sample size rationale has also been observed by others [3],[21],[68]. Further, we could not assess statistical power or effective sample size for many studies because of inadequate reporting of the number of candidate predictors.

In descriptions of participant selection, it often remained unclear whether participants were included in an unbiased way, notably with respect to refusals and whether all consecutive eligible participants were included. In contrast to randomized therapeutic trials, flow diagrams were hardly ever presented in prediction studies, which may reflect the difficulties of using these in prediction modelling studies because of the use of multiple analyses. The REMARK guidelines for prognostic tumour marker studies recommend using a REMARK profile table instead of a flow diagram [69].

Good reporting of how candidate predictors were pre-selected in our review compares favourably with other reviews [15],[20],[21],[29],[33],[35],[36],[48]. However, the methods used for further predictor selection during the statistical analyses were poorly reported. Univariable pre-selection and predictor selection in multivariable analyses based solely on *p*-values (with a <0.05 cut-off) was often used in predictor finding and model development studies. This approach may notably be problematic with low numbers of events and many candidate predictors. As the exact number of events per candidate predictor could almost never be assessed, it was not possible to determine whether reported results were indeed subject to overfitting or optimistic predictive performances. Several studies, however, did not rely exclusively on these methods for predictor selection, but rather also, as is recommended, included established predictors in their model regardless of statistical significance in their dataset.

Most studies reported the occurrence of missing data but did not report sufficient detail. Complete case analysis was by far the most commonly used approach to handle missing values, despite many methodological recommendations to do otherwise [10],[12],[13],[23],[24],[57],[58]. Recommended methods, such as multiple imputation, were applied and reported in very few studies, although this may be due to the fact that consensus in recommending these methods was arrived at only recently. As the reasons for missing values were insufficiently described in most studies that applied a complete case analysis, it was impossible to judge whether imputation methods would indeed have been appropriate.

Most studies correctly reported the (adjusted) predictor effects derived from the final multivariable analyses. Only a few studies also reported results of the univariable analyses, which is often a useful comparator. As noted in the REMARK guidelines [4], a comprehensive reporting of both univariable and multivariable analyses would allow readers to evaluate the adjustment for other predictors.

We observed much variation in the reporting of predictive performance measures. *C*-statistics or area under the ROC curves were the most frequently reported discrimination statistics, whereas measures of calibration (such as calibration slope) and overall measures of fit were rarely reported. Calibration measures are essential in model validation studies, to judge whether the predicted probabilities indeed match observed frequencies of the outcome under study.

The majority of model development studies reported predictive performance in the development data only. This apparent model performance, however, is generally too optimistic, as the model has been tailored to the dataset at hand. Overfitting is even more likely when the number of candidate predictors is large relative to the effective sample size [10],[12],[70]. The extent of this optimism may be estimated with so-called internal validation techniques [10],[12],[43],[70], but use of these techniques was rare. Similarly, only a very few model development studies reported an external validation of the model in the same paper. Accordingly, the generalisability of the performance of these reported models, especially in studies where prediction was the primary aim, is difficult to evaluate.

To further frame our results, a few issues need to be addressed. We examined prediction studies published in six high impact journals, likely representing higher quality studies. Reporting may have improved since 2008, although this is unlikely since no major reporting guidelines for this type of research have been produced recently. The recently published GRIPS statement and existing guidelines such as the REMARK guidelines, though focussed on specific types of studies, may improve reporting of future prediction research [4],[71]. We note that our work assessed researchers' reporting and statistical methods in modelling, and not necessarily the appropriateness of the design and conduct of the studies. Conduct of prediction research may be better than reported in the papers, since journals impose limits on the lengths of papers. It is important to note that a methodologically weak or statistically underpowered study is still a poor quality study, whether or not it is well reported. However, if it is poorly reported, then the reader will be unable to gauge its relevance and reliability.

To conclude, we identified poor reporting and poor methods in many published prediction studies, which limits the reliability and applicability of the published findings. However, encouraging findings included the frequent use of prospective studies, and adequate description of participant selection, predictor and outcome definitions, and the process for (pre)selection of candidate predictors. Improvement is clearly needed in blinding of assessment of outcomes for predictor information, many aspects of data analysis, the presentation of results of multivariable analyses, and the methods used to quantify and validate the predictive performance of a developed prediction model. Only a very small minority of the papers involved the most useful approaches in predicting participant clinical outcomes, namely, external validations or impact assessments of a previously developed prediction model.

## Supporting Information

Table S1 **Number of outcomes modelled, by type of prediction study.** All numbers are percentages, with absolute numbers in parentheses.
(DOC)

Text S1 **PRISMA checklist.**
(DOC)

Text S2 **Included studies.**
(DOC)
