# Supplementary material for: Reporting and Methods in Clinical Prediction Research: A Systematic Review
Source: PLoS Med. 2012 May 22;9(5):e1001221. doi: 10.1371/journal.pmed.1001221 (PMC3358324; doi:10.1371/journal.pmed.1001221)
Supplement: Table S1 — Number of outcomes modelled, by type of prediction study. All numbers are percentages, with absolute numbers in parentheses. (DOC) [file pmed.1001221.s001.doc]

Table S1. Number of outcomes modeled, by type of prediction study. All numbers are percentages and absolute numbers between parenthesis.

| **Type of prediction study** | **Total (N=70) ^a^** | **Predictor finding studies (N=50)** | **Development without external validation (N=11)** | **Development with external validation (N=3)** | **External validation without updating ^b^ (N=3)** | **Impact analysis ^b^ (N=3)** |
| --- | --- | --- | --- | --- | --- | --- |
| **Number of outcomes studied** |  |  |  |  |  |  |
| 1 | 60 (42) | 62 (31) | 73 (8) | 33 (1) | 67 (2) | 0 (0) |
| 2 | 26 (18) | 22 (11) | 18 (2) | 67 (2) | 33 (1) | 67 (2) |
| ≥ 3 | 14 (10) | 16 (8) | 9 (1) | 0 (0) | 0 (0) | 33 (1) |
| **Combined endpoint** ^c^ | 20 (14) | 18 (9) | 27 (3) | 67 (2) | 0 (0) | 0 (0) |

^a^ Unclear for 1 study

^b^ For external validation: the number of outcomes for which the accuracy of the prediction model was tested; for impact analysis: effect of prediction model on patient outcome, model consumer (physician) behavior or costs.

^c^ It was a combined endpoint if 1) the authors explicitly stated that they analyzed a combined endpoint and 2) when the predicted outcome was both fatal and nonfatal events. All cause death was not considered a combined endpoint
