# Supplementary material for: Reporting and Methods in Clinical Prediction Research: A Systematic Review
Source: PLoS Med. 2012 May 22;9(5):e1001221. doi: 10.1371/journal.pmed.1001221 (PMC3358324; doi:10.1371/journal.pmed.1001221)
Supplement: Text S2 — Included studies. (DOC) [file pmed.1001221.s003.doc]

1. Young Infants Clinical Signs Study Group (2008) Clinical signs that predict severe illness in children under age 2 months: a multicentre study. Lancet 371: 135-142.

2. Acharya CR, Hsu DS, Anders CK, Anguiano A, Salter KH, et al. (2008) Gene expression signatures, clinicopathological features, and individualized therapy in breast cancer. JAMA 299: 1574-1587.

3. Adabag AS, Therneau TM, Gersh BJ, Weston SA, Roger VL (2008) Sudden death after myocardial infarction. JAMA 300: 2022-2029.

4. Amin R, Widmer B, Prevost AT, Schwarze P, Cooper J, et al. (2008) Risk of microalbuminuria and progression to macroalbuminuria in a cohort with childhood onset type 1 diabetes: prospective observational study. BMJ 336: 697-701.

5. Bhattacharyya T, Nicholls SJ, Topol EJ, Zhang R, Yang X, et al. (2008) Relationship of paraoxonase 1 (PON1) gene polymorphisms and functional activity with systemic oxidative stress and cardiovascular risk. JAMA 299: 1265-1276.

6. Chan JA, Meyerhardt JA, Niedzwiecki D, Hollis D, Saltz LB, et al. (2008) Association of family history with cancer recurrence and survival among patients with stage III colon cancer. JAMA 299: 2515-2523.

7. Chan PS, Krumholz HM, Nichol G, Nallamothu BK (2008) Delayed time to defibrillation after in-hospital cardiac arrest. N Engl J Med 358: 9-17.

8. Cheyne H, Hundley V, Dowding D, Bland JM, McNamee P, et al. (2008) Effects of algorithm for diagnosis of active labour: cluster randomised trial. BMJ 337: a2396.

9. Dehghan A, Kottgen A, Yang Q, Hwang SJ, Kao WL, et al. (2008) Association of three genetic loci with uric acid concentration and risk of gout: a genome-wide association study. Lancet 372: 1953-1961.

10. Diep BA, Chambers HF, Graber CJ, Szumowski JD, Miller LG, Han LL, et al. (2008) Emergence of multidrug-resistant, community-associated, methicillin-resistant Staphylococcus aureus clone USA300 in men who have sex with men. Ann Intern Med 148: 249-257.

11. Fleming J, Brayne C (2008) Inability to get up after falling, subsequent time on floor, and summoning help: prospective cohort study in people over 90. BMJ 337: a2227.

12. Frank PI, Morris JA, Hazell ML, Linehan MF, Frank TL (2008) Long term prognosis in preschool children with wheeze: longitudinal postal questionnaire study 1993-2004. BMJ 336: 1423-1426.

13. Freiberg JJ, Tybjaerg-Hansen A, Jensen JS, Nordestgaard BG (2008) Nonfasting triglycerides and risk of ischemic stroke in the general population. JAMA 300: 2142-2152.

14. Gaziano TA, Young CR, Fitzmaurice G, Atwood S, Gaziano JM (2008) Laboratory-based versus non-laboratory-based method for assessment of cardiovascular disease risk: the NHANES I Follow-up Study cohort. Lancet 371: 923-931.

15. Gunnell D, Hawton K, Ho D, Evans J, O'Connor S, et al. (2008) Hospital admissions for self harm after discharge from psychiatric inpatient care: cohort study. BMJ 337: a2278.

16. Gutierrez OM, Mannstadt M, Isakova T, Rauh-Hain JA, Tamez H, et al. (2008) Fibroblast growth factor 23 and mortality among patients undergoing hemodialysis. N Engl J Med 359: 584-592.

17. Hall AJ, Logan JE, Toblin RL, Kaplan JA, Kraner JC, et al. (2008) Patterns of abuse among unintentional pharmaceutical overdose fatalities. JAMA 300: 2613-2620.

18. Head J, Ferrie JE, Alexanderson K, Westerlund H, Vahtera J, et al. (2008) Diagnosis-specific sickness absence as a predictor of mortality: the Whitehall II prospective cohort study. BMJ 337: a1469.

19. Henschke N, Maher CG, Refshauge KM, Herbert RD, Cumming RG, et al. (2008) Prognosis in patients with recent onset low back pain in Australian primary care: inception cohort study. BMJ 337: a171.

20. Hernandez AF, Shea AM, Milano CA, Rogers JG, Hammill BG, et al. (2008) Long-term outcomes and costs of ventricular assist devices among Medicare beneficiaries. JAMA 300: 2398-2406.

21. Hippisley-Cox J, Coupland C, Vinogradova Y, Robson J, Minhas R, Sheikh A, Brindle P (2008) Predicting cardiovascular risk in England and Wales: prospective derivation and validation of QRISK2. BMJ 336: 1475-1482.

22. Holtzer R, Verghese J, Wang C, Hall CB, Lipton RB (2008) Within-person across-neuropsychological test variability and incident dementia. JAMA 300: 823-830.

23. Imperiale TF, Glowinski EA, Lin-Cooper C, Larkin GN, Rogge JD, et al. (2008) Five-year risk of colorectal neoplasia after negative screening colonoscopy. N Engl J Med 359: 1218-1224.

24. Kahn SR, Shrier I, Julian JA, Ducruet T, Arsenault L, et al. (2008) Determinants and time course of the postthrombotic syndrome after acute deep venous thrombosis. Ann Intern Med 149: 698-707.

25. Kaklamani VG, Wisinski KB, Sadim M, Gulden C, Do A, et al. (2008) Variants of the adiponectin (ADIPOQ) and adiponectin receptor 1 (ADIPOR1) genes and colorectal cancer risk. JAMA 300: 1523-1531.

26. Kerr EA, Zikmund-Fisher BJ, Klamerus ML, Subramanian U, Hogan MM, et al. (2008) The role of clinical uncertainty in treatment decisions for diabetic patients with uncontrolled blood pressure. Ann Intern Med 148: 717-727.

27. Kruijshaar ME, Watson JM, Drobniewski F, Anderson C, Brown TJ, et al. (2008) Increasing antituberculosis drug resistance in the United Kingdom: analysis of National Surveillance Data. BMJ 336: 1231-1234.

28. Kuller LH, Tracy R, Belloso W, De Wit S, Drummond F, et al. (2008) Inflammatory and coagulation biomarkers and mortality in patients with HIV infection. PLoS Med 5: e203.

29. Laiyemo AO, Murphy G, Albert PS, Sansbury LB, Wang Z, et al. (2008) Postpolypectomy colonoscopy surveillance guidelines: predictive accuracy for advanced adenoma at 4 years. Ann Intern Med 148: 419-426.

30. Lederle FA, Larson JC, Margolis KL, Allison MA, Freiberg MS, et al. (2008) Abdominal aortic aneurysm events in the women's health initiative: cohort study. BMJ 337: a1724.

31. Limaye AP, Kirby KA, Rubenfeld GD, Leisenring WM, Bulger EM, et al. (2008) Cytomegalovirus reactivation in critically ill immunocompetent patients. JAMA 300: 413-422.

32. Lin GA, Dudley RA, Lucas FL, Malenka DJ, Vittinghoff E, et al. (2008) Frequency of stress testing to document ischemia prior to elective percutaneous coronary intervention. JAMA 300: 1765-1773.

33. Loeb M, Hanna S, Nicolle L, Eyles J, Elliott S, et al. (2008) Prognosis after West Nile virus infection. Ann Intern Med 149: 232-241.

34. Lyssenko V, Jonsson A, Almgren P, Pulizzi N, Isomaa B, et al. (2008) Clinical risk factors, DNA variants, and the development of type 2 diabetes. N Engl J Med 359: 2220-2232.

35. Marcucci G, Radmacher MD, Maharry K, Mrozek K, Ruppert AS, et al. (2008) MicroRNA expression in cytogenetically normal acute myeloid leukemia. N Engl J Med 358: 1919-1928.

36. McQueen MJ, Hawken S, Wang X, Ounpuu S, Sniderman A, et al. (2008) Lipids, lipoproteins, and apolipoproteins as risk markers of myocardial infarction in 52 countries (the INTERHEART study): a case-control study. Lancet 372: 224-233.

37. Meigs JB, Shrader P, Sullivan LM, McAteer JB, Fox CS, et al. (2008) Genotype score in addition to common risk factors for prediction of type 2 diabetes. N Engl J Med 359: 2208-2219.

38. Meltzer ME, Lisman T, Doggen CJ, de Groot PG, Rosendaal FR (2008) Synergistic effects of hypofibrinolysis and genetic and acquired risk factors on the risk of a first venous thrombosis. PLoS Med 5: e97.

39. Mermin J, Musinguzi J, Opio A, Kirungi W, Ekwaru JP, et al. (2008) Risk factors for recent HIV infection in Uganda. JAMA 300: 540-549.

40. Merritt WM, Lin YG, Han LY, Kamat AA, Spannuth WA, et al. (2008) Dicer, Drosha, and outcomes in patients with ovarian cancer. N Engl J Med 359: 2641-2650.

41. Montalvo G, Avanzini F, Anselmi M, Prandi R, Ibarra S, Marquez M, et al. (2008) Diagnostic evaluation of people with hypertension in low income country: cohort study of "essential" method of risk stratification. BMJ 337: a1387.

42. Moylan CA, Brady CW, Johnson JL, Smith AD, Tuttle-Newhall JE, et al. (2008) Disparities in liver transplantation before and after introduction of the MELD score. JAMA 300: 2371-2378.

43. Nader PR, Bradley RH, Houts RM, McRitchie SL, O'Brien M (2008) Moderate-to-vigorous physical activity from ages 9 to 15 years. JAMA 300: 295-305.

44. Parikh NI, Pencina MJ, Wang TJ, Benjamin EJ, Lanier KJ, et al. (2008) A risk score for predicting near-term incidence of hypertension: the Framingham Heart Study. Ann Intern Med 148: 102-110.

45. Peacock WF, De Marco T, Fonarow GC, Diercks D, Wynne J, et al. (2008) Cardiac troponin and outcome in acute heart failure. N Engl J Med 358: 2117-2126.

46. Pearce A, Law C, Elliman D, Cole TJ, Bedford H (2008) Factors associated with uptake of measles, mumps, and rubella vaccine (MMR) and use of single antigen vaccines in a contemporary UK cohort: prospective cohort study. BMJ 336: 754-757.

47. Peberdy MA, Ornato JP, Larkin GL, Braithwaite RS, Kashner TM, et al. (2008) Survival from in-hospital cardiac arrest during nights and weekends. JAMA 299: 785-792.

48. Perel P, Arango M, Clayton T, Edwards P, Komolafe E, et al. (2008) Predicting outcome after traumatic brain injury: practical prognostic models based on large cohort of international patients. BMJ 336: 425-429.

49. Pischon T, Boeing H, Hoffmann K, Bergmann M, Schulze MB, et al. (2008) General and abdominal adiposity and risk of death in Europe. N Engl J Med 359: 2105-2120.

50. Rawstron AC, Bennett FL, O'Connor SJ, Kwok M, Fenton JA, et al. (2008) Monoclonal B-cell lymphocytosis and chronic lymphocytic leukemia. N Engl J Med 359: 575-583.

51. Righini M, Le Gal G, Aujesky D, Roy PM, Sanchez O, Verschuren F, et al. (2008) Diagnosis of pulmonary embolism by multidetector CT alone or combined with venous ultrasonography of the leg: a randomised non-inferiority trial. Lancet 371: 1343-1352.

52. Ro KE, Gude T, Tyssen R, Aasland OG (2008) Counselling for burnout in Norwegian doctors: one year cohort study. BMJ 337: a2004.

53. Sasson C, Hegg AJ, Macy M, Park A, Kellermann A, et al. (2008) Prehospital termination of resuscitation in cases of refractory out-of-hospital cardiac arrest. JAMA 300: 1432-1438.

54. Sattar N, McConnachie A, Shaper AG, Blauw GJ, Buckley BM, et al. (2008) Can metabolic syndrome usefully predict cardiovascular disease and diabetes? Outcome data from two prospective studies. Lancet 371: 1927-1935.

55. Schetter AJ, Leung SY, Sohn JJ, Zanetti KA, Bowman ED, et al. (2008) MicroRNA expression profiles associated with prognosis and therapeutic outcome in colon adenocarcinoma. JAMA 299: 425-436.

56. Schlenk RF, Dohner K, Krauter J, Frohling S, Corbacioglu A, Bullinger L, et al. (2008) Mutations and treatment outcome in cytogenetically normal acute myeloid leukemia. N Engl J Med 358: 1909-1918.

57. Sekhri N, Feder GS, Junghans C, Eldridge S, Umaipalan A, et al. (2008) Incremental prognostic value of the exercise electrocardiogram in the initial assessment of patients with suspected angina: cohort study. BMJ 337: a2240.

58. Smith GC, Celik E, To M, Khouri O, Nicolaides KH (2008) Cervical length at mid-pregnancy and the risk of primary cesarean delivery. N Engl J Med 358: 1346-1353.

59. Stern DA, Morgan WJ, Halonen M, Wright AL, Martinez FD (2008) Wheezing and bronchial hyper-responsiveness in early childhood as predictors of newly diagnosed asthma in early adulthood: a longitudinal birth-cohort study. Lancet 372: 1058-1064.

60. Steyerberg EW, Mushkudiani N, Perel P, Butcher I, Lu J, et al. (2008) Predicting outcome after traumatic brain injury: development and international validation of prognostic scores based on admission characteristics. PLoS Med 5: e165.

61. Tice JA, Cummings SR, Smith-Bindman R, Ichikawa L, Barlow WE, et al. (2008) Using clinical factors and mammographic breast density to estimate breast cancer risk: development and validation of a new predictive model. Ann Intern Med 148: 337-347.

62. Tyson JE, Parikh NA, Langer J, Green C, Higgins RD (2008) Intensive care for extreme prematurity--moving beyond gestational age. N Engl J Med 358: 1672-1681.

63. Tzemos N, Therrien J, Yip J, Thanassoulis G, Tremblay S, Jamorski MT, et al., Siu SC (2008) Outcomes in adults with bicuspid aortic valves. JAMA 300: 1317-1325.

64. van Veen M, Steyerberg EW, Ruige M, van Meurs AH, Roukema J, et al. (2008) Manchester triage system in paediatric emergency care: prospective observational study. BMJ 337: a1501.

65. Vestergaard M, Pedersen MG, Ostergaard JR, Pedersen CB, Olsen J, et al. (2008) Death in children with febrile seizures: a population-based cohort study. Lancet 372: 457-463.

66. Vidula H, Tian L, Liu K, Criqui MH, Ferrucci L, et al. (2008) Biomarkers of inflammation and thrombosis as predictors of near-term mortality in patients with peripheral arterial disease: a cohort study. Ann Intern Med 148: 85-93.

67. Viros A, Fridlyand J, Bauer J, Lasithiotakis K, Garbe C, et al. (2008) Improving melanoma classification by integrating genetic and morphologic features. PLoS Med 5: e120.

68. Wang NC, Maggioni AP, Konstam MA, Zannad F, Krasa HB, et al. (2008) Clinical implications of QRS duration in patients hospitalized with worsening heart failure and reduced left ventricular ejection fraction. JAMA 299: 2656-2666.

69. Xie J, Brayne C, Matthews FE (2008) Survival times in people with dementia: analysis from population based cohort study with 14 year follow-up. BMJ 336: 258-262.

70. Zethelius B, Berglund L, Sundstrom J, Ingelsson E, Basu S, et al. (2008) Use of multiple biomarkers to improve the prediction of death from cardiovascular causes. N Engl J Med 358: 2107-2116.

71. Zheng SL, Sun J, Wiklund F, Smith S, Stattin P, Li G, et al. (2008) Cumulative association of five genetic variants with prostate cancer. N Engl J Med 358: 910-919.
